# Metformin-Induced Acute Hepatitis

**DOI:** 10.7759/cureus.38908

**Published:** 2023-05-11

**Authors:** Arooj Mian, Baha Aldeen Bani Fawwaz, Gurdeep Singh, Aimen Farooq, Ayman Koteish

**Affiliations:** 1 Internal Medicine, AdventHealth Orlando, Orlando, USA; 2 Gastroenterology and Hepatology, AdventHealth Orlando, Orlando, USA

**Keywords:** metformin-induced lactic acidosis, diabetes, s: hepatotoxicity, hepatotoxicity, liver disease, hepatitis, idiosyncratic liver injury, metformin-induced hepatotoxicity, metformin-induced acute hepatitis, drug-induced liver injury (dili)

## Abstract

Metformin is considered an initial oral pharmacotherapy of choice for treating hyperglycemia in type 2 diabetes mellitus (T2DM). Although safe in the vast majority of the population, rare side effects will come to light as the prevalence of T2DM continues to rise. We present a rare case of metformin-induced hepatotoxicity and possibly the first reported case of dose-dependent metformin-induced hepatotoxicity. This case report aims to make clinicians aware of this infrequent yet significant adverse reaction that can arise with metformin therapy.

## Introduction

Metformin is a biguanide that lowers blood glucose levels by inhibiting hepatic gluconeogenesis and increasing insulin sensitivity. It is a first-line oral anti-diabetic agent used in the treatment of type 2 diabetes mellitus (T2DM). Metformin is generally well tolerated. The most common adverse reaction to metformin is gastrointestinal distress. However, rare but serious adverse reactions can also occur, including hypoglycemia, dehydration, and lactic acidosis [1,2]. Here, we report a case of clinically significant metformin-induced acute hepatitis. To date, only a few cases of this rare adverse reaction have been reported in the widespread use of metformin for several decades [3-6]. Furthermore, there are no reports of dose-dependent hepatotoxicity of metformin.

This article was previously presented as a meeting abstract at the 2022 ACG Annual Scientific Meeting on October 25, 2022.

## Case presentation

**Figure 1:**
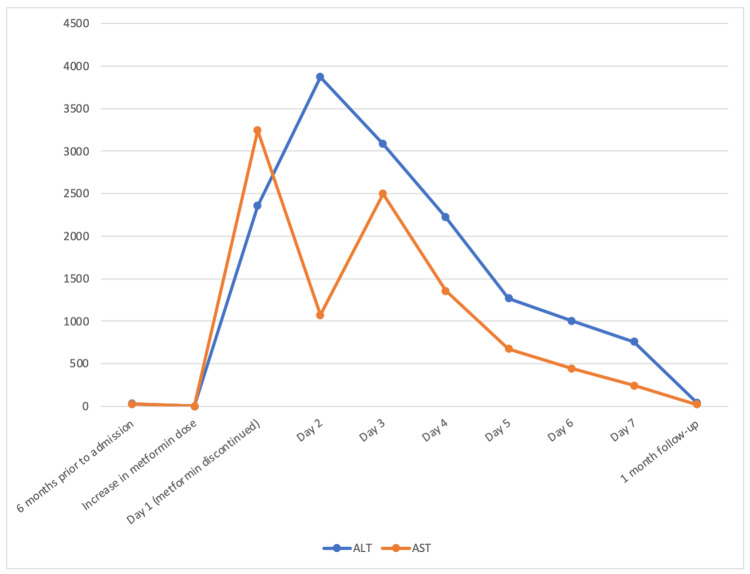
Dose-dependent metformin-induced hepatotoxicity. AST: Aspartate transaminase; ALT: Alanine aminotransferase.

A 75-year-old male with a past medical history of T2DM, hypertension, hyperlipidemia, and chronic heart failure presented with a one-month history of fatigue, nausea, vomiting, anorexia, and generalized abdominal pain. He denied current or previous symptoms of jaundice, ascites, hematemesis, and melena. The patient endorsed a prior history of moderate alcohol intake for several years but reported sobriety for the past five years. He denied the current or recent use of herbal products, dietary supplements, and exposure to toxins such as α-amanitin. Furthermore, he denied preexisting liver disease, prior abnormal liver enzymes, or a family history of liver disease. His home medications included aspirin 81 mg once daily, atorvastatin 40 mg once daily, bumetanide 2 mg once daily, metoprolol 100 mg once daily, and metformin 1000 mg twice daily. He denied the use of new prescription medications but noted a recent increase in his metformin dose from 500 mg to 1000 mg twice daily a few weeks prior to the presentation. He denied changes in the dosing or frequency of other home medications. His vital signs were stable on admission, and the physical examination revealed trace scleral icterus. Initial laboratory workup was remarkable for a hepatocellular pattern of liver injury (aspartate aminotransferase 3,241 units/L, alanine aminotransferase 2,355 units/L, alkaline phosphatase 170 units/L, total bilirubin 3.4 mg/dL, international normalized ratio 1.3, and serum albumin 2.66 g/dL). Other findings included an elevated serum creatinine level of 2 mg/dL, increased from a baseline level of 1 mg/dL, consistent with a pre-renal acute kidney injury likely secondary to dehydration. Workup to evaluate for the various etiologies of acute hepatitis was unremarkable and included acute viral hepatitis serologies (Hepatitis A, B, C, Epstein-Barr Virus, Cytomegalovirus, Herpes Simplex Virus, and HIV); metabolic liver diseases (α-1 antitrypsin, ceruloplasmin, copper); and autoimmune markers (antinuclear antibodies, anti-mitochondrial antibodies, anti-smooth muscle antibody, and anti-liver/kidney microsomal antibodies). A toxicology screen, including an acetaminophen level, was also negative. Furthermore, a phosphatidylethanol (PEth) test confirmed recent sobriety. Abdominal imaging with a Doppler ultrasound revealed patent hepatic vasculature, and computed tomography of the abdomen and pelvis was negative for signs suggestive of hepatic congestion, hepatic steatosis, portal hypertension, and cirrhosis. A liver biopsy was not performed due to the patient’s preference and the minimal impact it would have in guiding management. Given the negative workup above, the patient’s acute liver injury could not be explained by viral, alcoholic, ischemic, metabolic, or vascular insults. Instead, the pattern of liver injury was highly suspicious of drug-induced hepatitis resulting from increased metformin doses. It was postulated that an increase in the metformin dose resulted in lactic acidosis and, subsequently, acute hepatitis. Metformin was withdrawn. Subsequently, the patient’s liver enzymes gradually improved, and the presenting symptoms resolved. His renal function recovered with IV fluid hydration. He was then discharged in stable condition and advised to avoid metformin use in the future. The patient was followed up in the hepatology clinic four weeks after the hospital discharge and was found to be doing well with the normalization of the liver enzymes (Table 1, Figure 1). He has since been treated with sitagliptin and lifestyle modifications and has achieved good glycemic control.

**Table 1:** Pattern of liver enzyme elevation after an increase in metformin dose eight weeks prior to hospitalization with gradual return to baseline upon discontinuation of metformin.

	Alkaline phosphatase (40-129 units/L)	Alanine aminotransferase (4-51 units/L)	Aspartate aminotransferase (5-46 units/L)
Six months prior to admission	126	31	25
Hospital day 1	170	2,355	3,241
Hospital day 2	187	3,870	1,070
Hospital day 3	192	3,084	2,500
Hospital day 4	179	2,221	1,355
Hospital day 5	167	1,270	676
Hospital day 6	165	1,006	444
Hospital day 7	168	758	245
One month follow-up	131	42	20

## Discussion

Drug-induced liver injury (DILI) is a well-established problem and accounts for nearly 13% of all cases of acute liver failure in the United States [7]. Diagnosing DILI remains difficult as no specific serum biomarkers or tests are available to attribute the liver injury to a drug reliably. This can be especially challenging when hepatotoxicity is caused by a medication not considered intrinsically hepatotoxic such as metformin. Physicians may find clinical scales useful in the diagnostic process when faced with such a conundrum. The Naranjo Adverse Drug Reaction (ADR) Probability Scale was designed to help clinicians determine whether there is a causal relationship between a suspected drug and a clinical event. While this scale was not specifically developed to assess drug-induced hepatotoxicity, its simplicity has led to its wide use in assessing DILI [8]. When faced with the challenging diagnosis of DILI, clinicians may find it more useful to use the Roussel Uclaf Causality Assessment Method (RUCAM) scale. The RUCAM scoring system was specifically designed to help evaluate the likelihood of DILI [9]. In our case, the patient had an ADR probability scale of 7, indicating that the hepatotoxicity was probably due to metformin. To add consistency, the RUCAM scale was applied. Our patient scored 10 points, correlating with a high probability of metformin-induced hepatoxicity. In light of this, the patient’s liver injury was attributed to the recent increase in metformin therapy. Metformin is the first-line therapy in treating T2DM. Although considered safe, metformin may rarely cause severe hepatitis. The exact mechanism of metformin-induced hepatic injury is unknown. In our case, a diabetic patient presented with symptoms of acute hepatitis after an increase in metformin dose, the diagnosis of metformin-induced hepatotoxicity was supported by the causal relationship between an increase in the metformin dose and the onset of liver injury, exclusion of other causes of liver injury, recovery of liver function on discontinuation of metformin, and clinical scales with an ADR Probability Scale score of 7 and a RUCAM score of 10. With the rising burden of T2DM worldwide, rare side effects of commonly used anti-diabetic medications will continue to emerge. It is crucial for clinicians to familiarize themselves with these rare but serious adverse reactions. Through our case report, we aim to make clinicians aware of one such dose-dependent reaction of metformin-induced severe idiosyncratic acute liver injury.

## Conclusions

Our case indicates that patients can rarely develop hepatotoxicity after metformin use. Discontinuation of this offending agent allows for complete recovery of liver function. It is important for clinicians to familiarize themselves with this rare but serious adverse reaction of metformin therapy. 
